# How Informative are the Vertical Buoyancy and the Prone Gliding Tests to Assess Young Swimmers’ Hydrostatic and Hydrodynamic Profiles?

**DOI:** 10.2478/v10078-012-0020-x

**Published:** 2012-05-30

**Authors:** Tiago M. Barbosa, Mário J. Costa, Jorge E Morais, Marc Moreira, António J. Silva, Daniel A. Marinho

**Affiliations:** 1 Department of Sports Sciences, Polytechnic Institute of Bragança, Bragança, Portugal.; 2 Department of Sports Sciences, University of Trás-os-Montes and Alto Douro, Vila Real, Portugal.; 3 Department of Sports Sciences, University of Beira Interior, Covilhã, Portugal.; 4 Research Centre in Sports, Health and Human Development, Vila Real, Portugal.

**Keywords:** competitive swimming, evaluation, children, performance

## Abstract

The aim of this research was to develop a path-flow analysis model to highlight the relationships between buoyancy and prone gliding tests and some selected anthropometrical and biomechanical variables. Thirty-eight young male swimmers (12.97 ± 1.05 years old) with several competitive levels were evaluated. It were assessed the body mass, height, fat mass, body surface area, vertical buoyancy, prone gliding after wall push-off, stroke length, stroke frequency and velocity after a maximal 25 [m] swim. The confirmatory model included the body mass, height, fat mass, prone gliding test, stroke length, stroke frequency and velocity. All theoretical paths were verified except for the vertical buoyancy test that did not present any relationship with anthropometrical and biomechanical variables nor with the prone gliding test. The good-of-fit from the confirmatory path-flow model, assessed with the standardized root mean square residuals (SRMR), is considered as being close to the cut-off value, but even so not suitable of the theory (SRMR = 0.11). As a conclusion, vertical buoyancy and prone gliding tests are not the best techniques to assess the swimmer’s hydrostatic and hydrodynamic profile, respectively.

## Introduction

The identification of variables allowing predicting swimming performance is one of the main aims of the swimming “science” community (Barbosa et al., 2010c; Saavedra et al., 2010). It is consensual that it is possible to enhance performance manipulating biomechanical and/or energetics variables (Barbosa et al, 2010a). Anthropometrical and hydrodynamic variables are also described as being related to swimming performance (e.g, Geladas et al., 2005; Lätt et al., 2009a; 2009b; Silva et al., 2007). Anthropometrical, hydrodynamic and biomechanical testing procedures are often reported in the literature attempting to predict the swimming performance, as occurs in other competitive sports (e.g., Rogulj et al., 2009). Some programs for detection and follow-up of swimming talent, from children to adult/elite swimmer, include on regular basis such kind of tests (e.g., Hohmann and Seidel, 2010; Cazorla, 1993).

The swimmers hydrostatic profile (i.e., buoyancy force) can be measured with several validated techniques, as, for instance, the procedures reported by Yanai (2004) or Zamparo et al. (1996) based on hydrostatic weight lifting. The swimmers hydrodynamic profile (i.e., drag force) can also be assessed with other validated techniques, as, for instance, the velocity perturbation method (Marinho et al., 2010a; Kolmogorov and Duplishieva, 1992), the measuring active drag system (Hollander et al., 1986) or the swimmers’ passive drag assessment with the swimmer towing in water without segmental actions (Marinho et al., 2009; Pendergast et al., 2006). However, the procedures are expensive and require highly complex techniques to be used on daily basis by young swimmers coaches. That is the reason why young athlete’s coaches and researchers usually use cheaper and easier testing procedures for this and other assessments (e.g. Costa et al., 2009; Barbosa et al., 2010a). The vertical buoyancy test is one of those cheap and easy procedures to estimate the hydrostatic profile, as it is sometimes reported in the literature (e.g., Silva et al., 2007). The swimmer remains in a deep-water swimming pool, in the vertical position, in inspiratory apnea. The evaluator records with an ordinal scale the location of the water surface at a give head’s anatomical landmark. It is considered that larger portions of the swimmers body emerged represent a greater buoyancy capacity. To estimate the hydrodynamic profile, it is often used the prone gliding test. At least a couple of papers reported its use as well (Silva et al., 2007; Rama et al., 2006). In this test, swimmers are asked to push-off from the wall at a given immersion level and glide in the hydrodynamic position for the maximal horizontal distance that they are able to travel without limb movement. It is measured, in the swimming pool deck, the horizontal distance traveled by the swimmer until he stops gliding. It is considered that higher gliding distances represent the submission to lower drag force.

Both tests are used on regular basis because they are cheap and easy to apply as mentioned previously. To the best of our knowledge it is not reported in the literature any research about its validity and/or to which point vertical buoyancy and prone glide performances are informative of young swimmers’ hydrostatic and hydrodynamic profile, respectively. It seems possible that the vertical buoyancy and the prone gliding tests can be very rough estimations of the hydrostatic and hydrodynamic profiles. The vertical buoyancy adopts an ordinal scale that might not be representative of a continuous physical phenomenon. The prone gliding performance might depend not only from the drag force, but also from other variables such as the body height or the subject’s leg’s muscle power to push-off from the wall. So, some issues can be addressed regarding its validation and ecological data interpretation.

In competitive swimming: (i) anthropometrics influence hydrodynamics; (ii) hydrodynamics influence biomechanics; (iii) biomechanics influence the energetics and; (iv) energetics influences swimming performance. At least a couple of papers described the relationships between some selected anthropometrical and hydrodynamic variables (Barbosa et al., 2010b), as well as, between some selected biomechanical, energetics and swimming performance (Barbosa et al., 2010a). If vertical buoyancy and prone gliding tests are informative as they should be, it must present significant relationships with some anthropometrical and biomechanical variables. To clear out this, “Structural equation modeling” can be implemented for data analysis. This procedure consists of a mathematical approach for testing and estimating causal relationships using a combination of statistical data and qualitative causal assumptions previously defined by the researcher to be (or not to be) confirmed (Schreiber et al., 2006; Stein et al., 2012). This approach, rather than to identify variables, suggests the kind of interplay existing among them (Barbosa et al., 2010a). Instead of an exploratory data analysis, “Structural equation modeling” is considered as a confirmatory mathematical procedure. It uses various types of models to analyze relationships among observed variables, with the same basic goal of providing a quantitative test of a theoretical model hypothesized by a researcher. Although this approach is not very popular in the sport’s performance research, it is often used in other scientific domain (e.g., Crews and Losh, 1994; Kim and Cardinal, 2010). Hence, structural equating modeling allows analyzing the good-of-fit and the relationships between both hydrostatic and hydrodynamic tests and some selected anthropometrical and biomechanical variables. For the case of the theoretical backgrounds behind the vertical buoyancy test and the prone gliding test are correct, significant relationships between the tests and some anthropometrical and biomechanical variables must be verified.

Vertical buoyancy test attempts to estimate the buoyancy and the hydrostatic forces. From a mechanical point of view it is known that hydrostatic force is related to some anthropometrical variables. If the vertical buoyancy test attempts to estimate the hydrostatic forces, it has to be related with those anthropometrical variables as well. Otherwise, the test cannot be valid. The same idea supports the gliding test, attempting to be an estimation of the hydrodynamic forces. It is well know what are the variables affecting it. So, the gliding test has to be linked to those same variables to be considered as a valid procedure. If the vertical buoyancy and the prone gliding tests are rough estimations of the hydrostatic and hydrodynamic profiles then, probably those links would not be verified. So, it seems to exist a chance to checkout from an empirical point of view if those relationships actually exist or not.

Therefore, the aim of this research was to develop a path-flow analysis model to highlight the relationships between vertical buoyancy and prone gliding tests and selected anthropometrical and biomechanical variables. Since the theoretical backgrounds supporting the vertical buoyancy test and the prone gliding test have weaknesses and gaps, it was hypothesized that it could not be possible to establish significant relationships or links between some selected anthropometrical variables and vertical buoyancy and prone gliding performances and between these tests and biomechanical variables. Based on this rationality, probably the vertical buoyancy and prone gliding are not valid procedures to estimate the hydrostatic and the hydrodynamic profiles of a swimmer.

## Material and Methods

### Subjects

Thirty-eight young male swimmers (12.97 ± 1.05 years-old; Tanner stages 1–2) from several competitive levels were evaluated. Swimmers were engaged in competitive swimming at least for three years, having six to eight training sessions per week with one to two hours of training per session. All subjects participated on regular basis in Freestyle events at local, regional and/or national championships. Freestyle drills and training series represented almost a half of their training volume. The mean value for the best performance at the 200 [m] freestyle event in official short course competitions (at local, regional or national level) was 156.80 ± 17.30 [s] with a moderate-high dispersion between minimum and maximum data (130.27 ≤ *200 m freestyle* ≤ 206.27 s). For the structural equation modeling procedure that will be implemented, moderate-high data dispersion is required, including swimmers with a broad range of competitive levels.

Subjects and their legal tutors were informed of the potential experimental risks and signed an informed consent document prior to data collection. All procedures were in accordance to the Declaration of Helsinki in respect to Human research. The Institutional Review Board of the Polytechnic Institute of Bragança approved the study design.

### Anthropometrical data collection

For anthropometrical assessment the body mass, height, fat mass and body surface area were assessed. All measures were carried-out wearing a regular textile swimsuit. Body mass (BM) in [kg] was measured in the upright position with a digital scale (SECA, 884, Hamburg, Germany). Height (H) in [m] was measured in the anthropometrical position from vertex to the floor with a digital stadiometer (SECA, 242, Hamburg, Germany). Fat mass in [%] was estimated using a bio-impedance system (Tanita, BC-545, Middlesex, UK). Body surface area was estimated as being (Haycock et al., 1978):

(1) BSA=BM 0.5378×H 0.3964×0.024265

Where *BM* is the body mass in [kg] and *H* is the height converted to [cm]. An expert evaluator performed all anthropometrical measurements. Three measures of each variable were conducted and for further analysis the mean value was considered. Data reliability was very high (ICC = 0.96 ± 0.02).

### Hydrostatic and Hydrodynamic data collection

The hydrostatic and hydrodynamic performances were assessed with the vertical buoyancy and the prone gliding after wall push-off, respectively (Silva et al., 2007; Cazorla, 1993). In the vertical buoyancy test the swimmer was asked to be in the vertical position with the arms close to the trunk, in inspiratory apnea, without any movement and immersed in a 2.20 [m] deep swimming pool (Cazorla, 1993). The vertical buoyancy test takes approximately 30–60 seconds for the swimmer achieve a stable balance. The following discrete values were given when the water surface was by (Cazorla, 1993): (i) the neck (5 arbitrary units; a.u.); (ii) mouth (4 a.u.); (iii) nose (3 a.u.); (iv) eyes (2 a.u.) and; (v) vertex (1 a.u.). Whenever the water surface was in the middle of two anatomical landmarks, it was decided to select the one that was nearby.

In the prone gliding test, the subjects were asked to perform a maximal push-off from the wall at a deep of approximately 0.5 to 1.0 [m] in the first lane, with lane ropes separating it from the lateral wall and the second lane. Thereafter they should glide in the hydrodynamic position (head in neutral position, looking to the bottom of the swimming pool, legs fully extended and close together, arms fully extended at the front and with one hand above the other) with no limbs actions. Testing ended when swimmers achieved the water surface and/or were not able to make any further horizontal displacement of their body gliding and/or started any limb’s action. The maximal horizontal distance traveled by the swimmer from the wall to the feet was measured with a fiberglass tape (Nadic, Brebbia, Italy). An evaluator followed the swimmer during the trial closely to measure the distance. With the help of a vault it was recorded the maximum distance that the swimmer achieved after the wall push off parallel to the swimmer’s feet, tracing a perpendicular projection between the vault and the measuring tape.

Three measures of each hydrostatic and hydrodynamic variable were conducted. For further analysis the best performance of all three trials was considered.

### Biomechanical data collection

The swimming velocity, the stroke frequency and the stroke length were selected as biomechanical variables. Each swimmer performed a maximal 25 [m] front crawl swim with an underwater start. Subject performed the trial alone with no other swimmer in the same swim lane to reduce the drafting or pacing effects. The swimmers were advised to reduce gliding during the start. Swimming velocity was measured in the middle 15 [m] of the swimming pool as:

(2) $$\bar{\text{v}}=\frac{\text{d}}{\text{t}}$$

Where *v* is the mean swimming velocity in [m.s^−1^], *d* is the distance covered by the swimmer in [m] and, *t* is the time spent to cover such distance in [s] measured with a chronometer (Golfinho Sports MC 815, Aveiro, Portugal) by an expert evaluator. The stroke frequency (SF) was measured with a chrono-frequency meter (Golfinho Sports MC 815, Aveiro, Portugal) from three consecutive stroke cycles, in the middle of the 15 [m] distance of the swimming pool, by an expert evaluator as well. Stroke length (SL) in [m] was estimated as (Craig and Pendergast, 1979):

(3) $$\text{SL}=\frac{\bar{\text{v}}}{\text{SF}}$$

### Theoretical model

**Figure 1:**
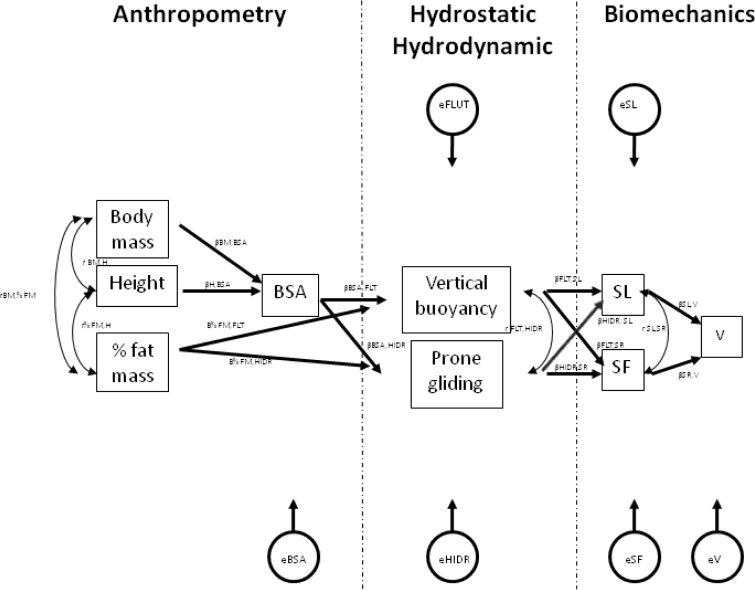
*Theoretical path-flow model. BSA – body surface area; SL – stroke length; SF – stroke frequency; v – swimming velocity; βx_i_,y_i_– beta value for regression model between exogenous (x_i_) and endogenous (y_i_) variables; ex_i_– disturbance term for a given endogenous variable; rx_i_,y_i_– correlation coefficient between two variables; x_i_→y_i_– variable y_i_depends from variable(s) x_i_; x_i_↔y_i_variable y_i_is associated to variable x_i_*.

The theoretical model (Figure 1) was developed according to main papers regarding to the relationships between anthropometrics, hydrodynamics and biomechanics variables (e.g. Barbosa et al., 2010a; 2010b; 2010c; Lavoie and Montpetit, 1986) and the assessments included in some programs for detection and follow-up of swimming talents (e.g., Silva et al., 2007; Cazorla, 1993; Saavedra et al., 2010).

It is considered that the anthropometrics domain will influence the hydrostatic/hydrodynamic domain and the last one will influence the biomechanics domain (Barbosa et al., 2010a; 2010b). Some anthropometrical variables that are reported on regular basis in swimming literature, such as the body mass, height, fat mass and body surface area were selected (e.g., Mazza et al., 1994; Geladas et al., 2005; Jagomägi and Jürimäe, 2005; Lätt et al., 2009a; 2009b). Vertical buoyancy and prone gliding tests were adopted because the main focus of this paper was to understand its validity, as well as, its relationship with anthropometrical and biomechanical variables. Regarding to biomechanical variables, the stroke length, stroke frequency and swimming velocity were assessed (e.g., Barbosa et al., 2010a; 2010c). It seems to exist a co-variation between the body mass, the height, the fat mass and the body surface area (e.g., Haycock et al., 1978). Both the fat mass and the body surface area will influence the vertical buoyancy and the prone gliding performances (Costill et al., 1992). There will be a co-variation between vertical buoyancy and prone gliding. Both tests will interplay with the stroke frequency and the stroke length. The stroke frequency and stroke length will influence swimming velocity (Craig and Pendergast, 1979; Keskinen et al. 1989).

### Statistical procedures

The normality and homocedasticity assumptions were verified with the Shapiro-Wilk and the Levene tests, respectively. Descriptive statistics (maximum, minimum, mean, one standard deviation, coefficient of variation) from all variables were calculated (Hopkins et al., 2009). Pearson’s correlation coefficients were computed between buoyancy and prone gliding tests and remaining selected variables. As a rule of thumb, Pearson’s correlation coefficients between: (i) 0.00 ≤*r*< 0.30 were considered weak; (ii) 0.30 ≤*r*< 0.70 were considered moderate and; (iv) 0.70 ≤*r*≤ 1.00 were considered high. The statistical significance was set at *p*≤ 0.05.

Path-flow analysis was performed with the estimation of standardized linear regression coefficients between the exogenous and endogenous variables. All assumptions to perform the path-flow analysis were considered. When appropriate, according to the theoretical model, simple or multiple linear regression models were computed. Standardized regression coefficients (β) were considered. Significance of each β was assessed with the t-Student test (p ≤ 0.05). The effect size of the disturbance term, reflecting unmeasured variables, for a given endogenous variable, was 1−*r*^2^.

To measure the quality of the model fit, the standardized root mean square residuals (SRMR) was computed:

(6) $$\mathrm{SRMR}=\sqrt{\frac{\sum_{i=1}^{p}\sum_{i=1}^{q}{\left(rij-pij\right)}^{2}}{p+q}}$$

Where ρ_ij_ are the correlations predicated on the model, estimated by the sum of the casual associations (direct effects and indirect effects) with the non-casual association (non-estimate effects plus spurious effects) and *r_ij_* are the observed correlations between *p+q* variables. *SRMR* measures the standardized difference between the observed covariance and predicted covariance. It is considered qualitatively if: (i) *SRMR*< 0.1 that the model adjust to the theory; (ii) *SRMR*< 0.05 that the model adjusts very well to the theory and; (iii) *SRMR* ∼ 0 that the model is perfect (adapted from Hu and Bentler, 1999).

## Results

Table 1 presents the descriptive statistics from all selected variables. Data dispersion, expressed as 1 *SD,* was moderate-high for most variables. The same phenomena can be verified analyzing other dispersion statistics such as the data range or the coefficient of variation. The higher data range was verified in the vertical buoyancy (1.00 ≤ *vertical buoyancy* ≤ 3.00 a.u.; CV = 39.7%), in the fat mass (7.7 ≤ *fat mass* ≤ 28.2 %; CV = 33.2%) and in the body mass (32.3 ≤ *body mass* ≤ 68.6 kg; CV = 26.4%).

Table 2 presents the Pearson’s correlation coefficients between the vertical buoyancy performance and the prone gliding performance with remaining variables. No significant associations were found between vertical buoyancy performance and any of the selected variables. On the other hand, all variables presented significant association with the prone gliding performance, except for the *v*. For the significant associations, Pearson’s correlation coefficients ranged between moderate (e.g. r*_pronegliding,SF_* = −0.54; p < 0.01) and high (e.g. r_pronegliding,BSA_ = 0.75; p < 0.001) associations.

**Figure 2:**
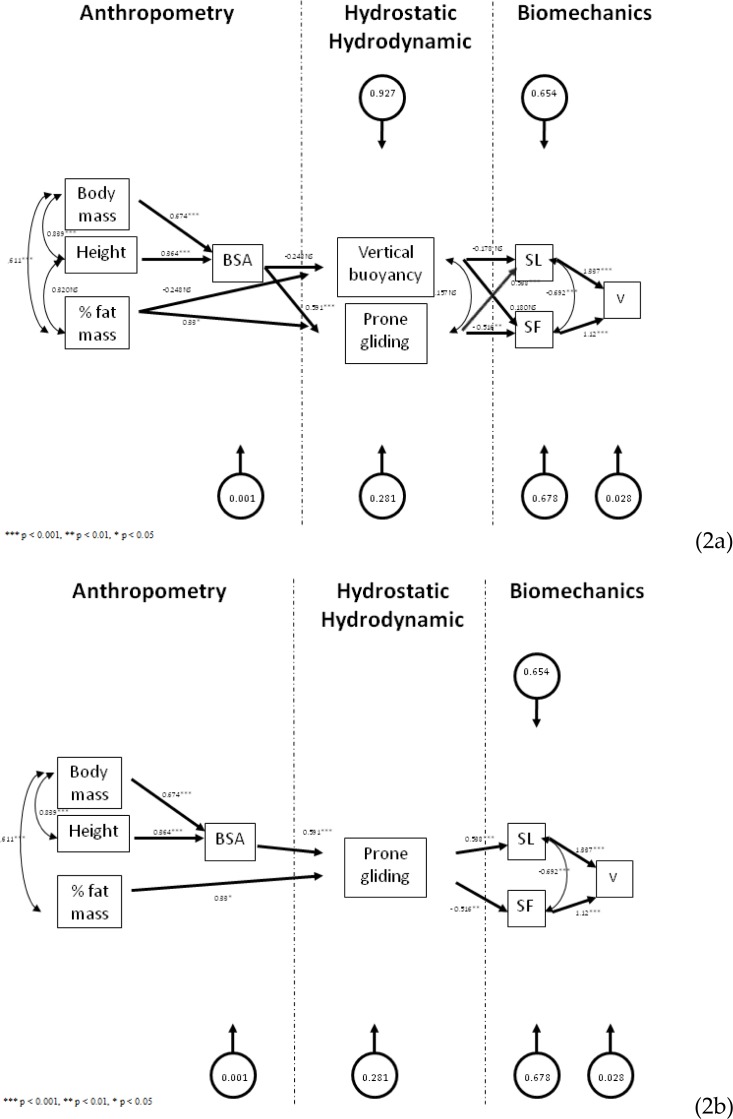
*Confirmatory path-flow models including non-significant paths (2a) and deleting non-significant paths with subsequent re-computation of remain data (2b). BSA – body surface area; SL – stroke length; SF – stroke frequency; v – swimming velocity; x_i_→y_i_– variable y_i_depends from variable(s) x_i_; x_i_↔y_i_variable x_i_is associated to variable y_i_*.

Figure 2 presents the confirmatory path-flow models. A couple of partial relationship (i.e., theoretical paths) did not confirm the hypothesis (Figure 2a). The confirmatory model excluded all paths linking to the vertical buoyancy (β*_BSA,vertical,buoyancy_* = −0.242, p > 0.05; β*_fat mass,vertical,buoyancy_* = −0.248, p > 0.05; β*_vertical buoyancy,SL_* = −0.178, p > 0.05; β*_vertical buoyancy,SF_* = 0.180, p > 0.05) and the relationship between height and fat mass (r*_height,fat mass_* = 0.32, p > 0.05). The *v* had a 97.2% capability to be predicted based on the *SF* and the *SL*. However, based on the prone gliding performance, only 32.2% from the *SF* and 34.6% from the *SL* were predicted (Figure 2a). Deleting the vertical buoyancy from the model and re-computing the data again, does not lead to changes in the prediction level (Figure 2b).

Regarding the good-of-fit from the confirmatory model, after deleting the non-significant paths, the *SRMR* was very close to the selected cut-off value. Even so, from a qualitative point of view the model was considered as not suitable of the theory (*SRMR* = 0.11). In this sense the removal of the vertical buoyancy had a major impact in the model’s quality.

## Discussion

The purpose of this paper was to develop a path-flow analysis model to highlight the relationships between vertical buoyancy and prone gliding tests and some selected anthropometrical and biomechanical variables. Authors aimed to verify if both tests are valid and informative of the swimmers hydrostatic/hydrodynamic profile. The confirmatory model excluded the vertical buoyancy and the relationship between height and fat mass. Deleting the vertical buoyancy test had a major impact in the model’s good-of-fit.

Descriptive statistics (central and dispersion parameters) of the variables selected are slightly within the range of values reported in the literature for swimmers from similar cohort group (i.e., same gender, chronological and biological age) (e.g., Barbosa et al., 2010a; Schidt and Ungerechts, 2008; Silva et al., 2007; Jürimäe et al., 2007; Greco et al., 2005; Lätt et al., 2009a). To compute the Structural equation modeling procedure moderate-high data dispersion is required. Since a broad data dispersion is verified (e.g. standard deviation, data range, coefficient of variation) it is possible to understand the behavior of the selected variables in a wide range of performance level.

Non-significant associations were found between vertical buoyancy test and selected variables. On the other hand, several selected variables presented significant and moderate-high associations with prone gliding test. Increasing prone gliding distance was related with higher *BM*, *H*, *fat mass*, *BSA, SL* and lower *SF*. So, data suggests the existence of some kind of relationship between the hydrodynamic domain and the anthropometric and the biomechanics domains. Therefore, the theoretical model path-flow designed, where fluid mechanics variables depend on anthropometrics and influence biomechanics, might be correct (Barbosa et al., 2010b).

Regarding the theoretical and the confirmatory models, two partial relationships (i.e., theoretical paths) did not confirm the hypothesis. The confirmatory model excluded the vertical buoyancy and the relationship between height and fat mass. The *v* had a high capability to be predicted based on the *SF* and the *SL* (i.e., 97.2%). Such relationship is consensual in the literature (Barbosa et al., 2010a; Craig and Pendergast, 1979; Keskinen et al., 1989). Even so, some multicolinearity phenomena might be involved as *SL* was estimated with equation 3. Only 32.2% and 34.6 % of the *SF* and *SL,* respectively, were predicted based on the prone gliding performance. Some hydrostatic and/or hydrodynamic variables that are not included in the model might have a significant influence in both biomechanical variables. Adding other variables that are measured and not estimated (e.g., frontal surface area, projected frontal area, active drag or passive drag) (Pendergast et al., 2006; Yanai, 2004; Zamparo et al., 1996; Kolmogorov and Duplishieva, 1992; Hollander et al., 1986) could increase the model’s prediction level. Those procedures are complex, time consuming and expensive. Therefore, such techniques are not used on regular basis by young swimmer’s coaches. Young swimmer’s coaches prefer to use the vertical buoyancy test and the prone gliding test.

Indeed, a couple of issues confirm our hypothesis that probably both tests are not valid and informative as it is considered: (i) the vertical buoyancy test was completely removed from the model and; (ii) there was a low capacity to predict *SF* and *SL* based on the prone gliding performance. Buoyancy is an external force with a vertical and upright direction, opposite to the body’s weight that is computed as:

(7) B=ρ⋅g⋅V

Where *B* is the buoyancy force in [N], *ρ* is the density of the water in [kg.m^−3^], *g* is the gravitational acceleration in [m.s^−2^] at the location in question and *V* is the volume of the displaced body of the liquid in [m^3^]. From a theoretical point of view, it is considered that during the vertical buoyancy test the swimmer’s body is in a fluid mechanics statics situation, where the net forces are:

(8) $$\sum F_{i}=0$$

Being considered as F_i_:

(9) W+B=0

Where *W* is the bodies weight force in [N] and *B* the buoyancy force in [N]. So:

(10) (m⋅g)+(ρ⋅g⋅V)=0

Otherwise, if the swimmer’s buoyancy force exceeds its weight, he will rise in the water and emerge. On the other hand, if the swimmer’s weight exceeds its buoyancy he will sink. The arbitrary units scale (a.u.) used in the vertical buoyancy scale test tries to be an ordinal measure of this physics phenomenon. As any ordinal measure, the scale used describes a ranking order, instead of a relative size or degree of difference between the items measured. It is possible to exist some major limitations using an ordinal scale to measure this physical phenomenon. Therefore, the ordinal scale should be changed to an interval scale.

Some physiological and anthropometrical variables are related to the buoyancy force. There are several reports in the literature describing that buoyancy is related to the body composition (Mazza et al., 1994). Theoretical and experimental studies report that a higher percentage of fat mass imposes higher buoyancy (Zamparo et al., 1996; Mazza et al., 1994; Costill et al., 1992). In addition, a higher buoyancy capacity seems to be related to a better horizontal body position and a lower drag force (Yanai, 2004; Zamparo et al., 1996). Even so, the co-variation between the vertical buoyancy and the prone gliding test was not significant. The relationship was negative rather than being positive. Respiratory variables (e.g. lungs volume, vital capacity, residual volume and tidal volume) might also play a significant influence in the vertical buoyancy performance. So, some weaknesses can be addressed in the physics background supporting the vertical buoyancy test concept. New researches comparing and validating the vertical buoyancy test with some specific gold-standard test should be conducted in the future to clear out this issue.

Prone gliding test is used as an estimation of the swimmer’s passive drag. Drag force is computed as:

(11) $$D=\frac{1}{2}\cdot \rho \cdot v^{2}\cdot S\cdot c_{d}$$

Where *D* is the drag force in [N], *ρ* is the density of the water in [kg.m^−3^], *v* is the swimming velocity in [m.s^−1^], *S* is the projected frontal surface area of the swimmers in [cm^2^] and *Cd* is the drag coefficient [dimensionless] (changing owning to shape, orientation and Reynolds number). Thus, it is considered that as hydrodynamic is the swimmer’s position, the lower is the drag force the swimmer is submitted to. However, the prone gliding test does not consider a couple of variables that might imposes some bias: (i) drag force is dependent from the swimmer’s immersion depth (Marinho et al., 2010b; Pease and Venell, 2010); (ii) the gliding distance is related not only to drag force, but also to propulsive forces such as the leg’s muscle power and/or neuromuscular patterns (Miriam and Dietmar, 2003) and/or the reaction forces (Pereira et al., 2006) during the wall push-off; (iii) when gliding close to the water surface drag force is also dependent from the Froude number, which is related to the body’s length (Kjendlie and Stallman, 2008). In this sense, some precaution should also be taken interpreting the prone gliding test performance as it can have some bias.

Confirmatory path-flow model can be considered as very close to the *SRMR* cut-off value adopted. Even so, from a qualitative point of view, the model cannot be considered as suitable of the theory (*SRMR* = 0.11). The model’s good-of-fit is related to the delete of all paths linking vertical buoyancy with remaining anthropometrical and biomechanical variables. Four paths were deleted: (i) *BSA* – vertical buoyancy; (ii) vertical buoyancy – *SL*; (iii); vertical buoyancy – *SF* and; (iv) vertical buoyancy – prone gliding. The *SRMR* measures the standardized difference between the observed covariance and the predicted covariance. Therefore, deleting the four paths increased the *SRMR* value, going it beyond the quality cut-off value considered for 0.01 units.

It should be once again stressed out that the current study aimed to understand to which point the vertical buoyancy and the prone gliding tests, used on regular basis to assess young swimmers, are informative of the subjects’ fluid mechanics profile. Moreover, this study aimed to analyze if the tests present any relationship with anthropometrics and biomechanics as theoretical background pointed out. Based on current data, it seems that the vertical buoyancy and the gliding tests do not depend exclusively from buoyancy force and drag force, respectively. That is the reason why both cannot be considered as valid testing procedures to estimate the hydrostatic and hydrodynamic profile. It can be addressed as main limitations of the study: (i) the need to identify, in a near future, through exploratory data analysis procedures other variables that influence the prone gliding performance, such as, as hypothesis, the leg’s muscle power, trunk transverse surface area, etc.; (ii) the final model obtained might not be representative of what occurs in other cohort groups (i.e., female swimmers or adult/elite swimmers); (iii) a “classical” validation procedure was not carried-out to quantify the accurate value of over and/or underestimation that each test presents in comparison with hydrostatic and hydrodynamic gold-standard techniques.

As a conclusion, the vertical buoyancy test did not present any relationship with anthropometrical and biomechanical variables nor with the prone gliding test as suggested by its theoretical background. Prone gliding performance depends not only on drag force. The low prediction level of the prone gliding test might be related to other variables that are not considered in its theoretical background and, therefore, were not included in the model. Hence, some precaution should be taken interpreting data from the prone gliding test. As a coach-friendly conclusion, vertical buoyancy and prone gliding tests are easy and cheap procedures. However, they are not the best techniques to assess the swimmer’s hydrostatic and hydrodynamic profile, respectively.

## Figures and Tables

**Table 1:** Descriptive statistics for anthropometrical, hydrodynamic and biomechanical variables

	Mean (n = 38)	1 SD	Max	Min
Body mass [kg]	50.4	13.3	68.6	32.3
Height [m]	1.59	0.12	1.68	1.36
Fat mass [%]	14.9	4.95	28.2	7.70
BSA [m^2^]	1.49	0.23	2.02	1.16
Vertical buoyancy [a.u.]	1.31	0.52	3.00	1.00
Prone gliding [m]	6.81	0.79	8.20	5.50
SL [m]	1.64	0.20	2.14	1.25
SF [Hz]	0.89	0.08	1.03	0.69
v [m.s^−1^]	1.46	0.13	1.69	1.15

**Table 2:** Pearson’s Correlation matrix between hydrostatic and hydrodynamic tests with remain variables

	Vertical Buoyancy (n = 38)	Prone Gliding (n = 38)
BM	−0.19 (p = 0.28)	0.74 (p < 0.001)
H	−0.27 (p = 0.12)	0.68 (p < 0.001)
Fat mass	−0.32 (p = 0.09)	0.72 (p < 0.001)
BSA	−0,21 (p = 0.22)	0.75 (p < 0.001)
SL	−0.25 (p = 0.15)	0.56 (p = 0.001)
SF	0.23 (p = 0.19)	−0.54 (p = 0.001)
v	−0.08 (p = 0.66)	0.15 (p = 0.40)

## References

[b1-jhk-32-21] Barbosa TM, Costa MJ, Coelho J, Moreira M, Silva AJ (2010a). Modeling the links between young swimmer’s performance: energetic and biomechanics profile. Pediatr Exerc Sci.

[b2-jhk-32-21] Barbosa TM, Costa MJ, Marques MC, Silva AJ, Marinho DA (2010b). A model for active drag force exogenous variables in young swimmers. J Hum Sports Exerc.

[b3-jhk-32-21] Barbosa TM, Bragada JA, Reis VM, Marinho DA, Carvalho C, Silva AJ (2010c). Energetics and biomechanics as determining factors of swimming performance: updating the state of the art. J Sci Med Sports.

[b4-jhk-32-21] Cazorla G (1993). Tests spécifiquesd’évaluation du nager.

[b5-jhk-32-21] Costa AM, Silva AJ, Louro H, Reis VM, Garrido ND, Marques MC, Marinho DA (2009). Can the curriculum be used to estimate critical velocity in young competitive swimmers?. J Sports Sci Med.

[b6-jhk-32-21] Costill D, Maglischo E, Richardson A (1992). Swimming.

[b7-jhk-32-21] Craig A, Pendergast D (1979). Relationships of stroke rate, distance per stroke and velocity in competitive swimming. Med Sci Sports Exerc.

[b8-jhk-32-21] Crews DE, Losh S (1994). Structural modeling of blood pressure in Samoans. CollAntropol.

[b9-jhk-32-21] Geladas ND, Nassis GP, Pavlicevic S (2005). Somatic and physical traits affecting sprint swimming performance in young swimmers. Int J Sports Med.

[b10-jhk-32-21] Greco CC, Denadai B (2005). Critical speed and endurance capacity in young swimmers: effects of gender and age. PediatrExercSci.

[b11-jhk-32-21] Haycock GB, Schwartz GJ, Wisotsky DH (1978). Geometric method for measuring body surface area: a height-weight formula validated in infants, children, and adults. J Pediatr.

[b12-jhk-32-21] Hohmann A, Seidel I, Kjendlie PL, Stallman RK, Cabri J (2010). Talent prognosis in young swimmers. Biomechanics and Medicine in Swimming XI.

[b13-jhk-32-21] Hollander AP, de Groot G, van IngenSchenau G, Toussaint H, de Best H, Peeters W, Meulemans A, Schreurs A (1986). Measurement of active drag during crawl stroke swimming. J Sports Sci.

[b14-jhk-32-21] Hopkins WG, Marshall SW, Batterham AM, Hanin J (2009). Progressive statistics for studies in sports medicine and exercise science. Med Sci Sports Exerc.

[b15-jhk-32-21] Hu L, Bentler PM (1999). Cutoff criteria for fit indexes in covariance structure analysis: Conventional criteria versus new alternatives. Structural Eq Model.

[b16-jhk-32-21] Jagomägi G, Jürimäe T (2005). The influence of anthropometrical and flexibility parameters on the results of breaststroke swimming. AnthropolAnz.

[b17-jhk-32-21] Jürimäe J, Haljaste K, Cichella A, Lätt E, Purge P, Leppik A, Jürimäe T (2007). Analysis of swimming performance from physical, physiological and biomechanical parameters in young swimmers. PediatrExercSci.

[b18-jhk-32-21] Keskinen KL, Till LJ, Komi PV (1989). Maximum velocity swimming: interrelationship of stroke characteristics, force production and anthropometric variables. Scan J Med Sci Sports.

[b19-jhk-32-21] Kim YH, Cardinal BJ (2010). Psychosocial correlates of Korean adolescents’ physical activity behavior. J Exerc Sci Fit.

[b20-jhk-32-21] Kjendlie PL, Stallman (2008). Drag characteristics of competitive swimming children and adults. J ApplBiomech.

[b21-jhk-32-21] Kolmogorov S, Duplishcheva O (1992). Active drag, useful mechanical power output and hydrodynamic force in different swimming strokes at maximal velocity. J Biomech.

[b22-jhk-32-21] Lätt E, Jürimäe J, Haljaste K, Cicchella, Jürimäe T (2009a). Longitudinal development of physical and performance parameters during biological maturation of young male swimmers’. Percept Motor Skills.

[b23-jhk-32-21] Lätt E, Jürimäe J, Haljaste K, Cicchella A, Purge P, Jürimäe T (2009b). Physical Development and Swimming Performance During Biological Maturation in Young Female Swimmers. Coll Antropol.

[b24-jhk-32-21] Lavoie JM, Montpetit R (1986). Applied Physiology of swimming. Sports Med.

[b25-jhk-32-21] Marinho DA, Barbosa TM, Klendlie P-L, Vilas-Boas JP, Alves FB, Rouboa AI, Silva AJ, Peter M (2009). Swimming Simulation. Computational Fluid Dynamics for sport simulation.

[b26-jhk-32-21] Marinho DA, Garrido N, Barbosa TM, Reis VM, Silva AJ, Costa AM, Marques MC (2010a). Can 8 weeks of training in female swimmers affect active drag?. J Sports Sci Med.

[b27-jhk-32-21] Marinho DA, Barbosa TM, Mantripragada N, Vilas-Boas JP, Rouard AI, Msantha VR, Rouboa AI, Silva AJ, Kjendlie PL, Stallman RK, Cabri J (2010b). The gliding phase in swimming: the effect of water depth. In: Biomechanics and Medicine in Swimming XI.

[b28-jhk-32-21] Mazza J, Ackland TR, Bach T, Cosolito P, Carter L, Ackland TR (1994). Absolute body size. Kineanthropometry in Aquatic Sports.

[b29-jhk-32-21] Miriam R, Dietmar S, Muller E, Schwmeder H, Zallinger G, Fastenbauer V (2003). The influence of stretch-shortening-cycle (SSC) on turning performance in competition swimmers. Proceedings of the 8th Annual Congress of the European College of Sport Science.

[b30-jhk-32-21] Pease DL, Vennell R, Kjendlie PL, Stallman RK, Cabri J (2010). The effect of angle of attack and depth on passive drag. Biomechanics and Medicine in Swimming XI.

[b31-jhk-32-21] Pendergast DR, Capelli C, Craig AB, di Prampero PE, Minetti AE, Mollendorf J, Termin II, Zamparo P, Vilas-Boas JP, Alves F, Marques A (2006). Biophysics in swimming. Biomechanics and Medicine in Swimming X.

[b32-jhk-32-21] Pereira S, Araújo L, Freitas E, Gatti R, Silveira G, Roesler H, Vilas-Boas JP, Alves F, Marques A (2006). Biomechanical analysis of the turn in front crawl swimming. Biomechanics and Medicine in Swimming X.

[b33-jhk-32-21] Rama L, Santos J, Gomes P, Alves F, Vilas-Boas JP, Alves F, Marques A (2006). Determinant factors related to performance in young swimmers. Biomechanics and Medicine in Swimming X.

[b34-jhk-32-21] Rogulj N, Papic V, Cavala M (2009). Evaluation models of some morphological characteristics for talent scouting in sport. Coll Antropol.

[b35-jhk-32-21] Saavedra JM, Escalante Y, Rodríguez FA (2010). A multivariate analysis of performance in young swimmers. PediatrExercSci.

[b36-jhk-32-21] Schidt A, Ungerechts BE, Nomura T, Ungerechts BE (2008). The effect of cognitive intervention on stroke distance in age-group swimmers. The Book of Proceedings of the 1st International Scientific Conference of Aquatic Space Activities.

[b37-jhk-32-21] Schreiber JB, Nora A, Stage FK, Barlow EA, Jamie King (2006). Reporting structural equation modeling and confirmatory factor analysis results: a review. J Educ Res.

[b38-jhk-32-21] Silva AJ, Costa AM, Oliveira PM, Reis VM, Saavedra J, Perl J, Rouboa A, Marinho DA (2007). The use of neural network technology to model swimming performance. J Sports Sci Med.

[b39-jhk-32-21] Stein CM, Morris NJ, Nock NL (2012). Structural equation modeling. Methods MolBiol.

[b40-jhk-32-21] Yanai T (2004). Buoyancy is the primary source of generating bodyroll in front-crawl swimming. J Biomech.

[b41-jhk-32-21] Zamparo P, Antonutto G, Capelli C, Francescato M, Girardis M, Sangoi R, Soule R, Pendergast D (1996). Effects of body density, gender and growth on underwater torque. Scan J Med Sci Sports.

